# Upregulation of *LncRNA‐HIT* promotes migration and invasion of non‐small cell lung cancer cells by association with ZEB1

**DOI:** 10.1002/cam4.948

**Published:** 2016-10-27

**Authors:** Xiaojing Jia, Zhicheng Wang, Ling Qiu, Yanming Yang, Yunlong Wang, Zhishen Chen, Zhongshan Liu, Lei Yu

**Affiliations:** ^1^ Department of RadiotherapyThe Second Hospital of Jilin UniversityChangchun130041China; ^2^ Key Laboratory of RadiobiologyMinistry of HealthSchool of Public HealthJilin UniversityChangchun130021China

**Keywords:** Invasion, lncRNA, *lncRNA‐HIT*, migration, non‐small cell lung cancer, ZEB1

## Abstract

Lung cancer is the most common solid tumor and the leading cause of cancer‐related mortality worldwide. Non‐small cell lung cancer (NSCLC) accounts for approximately 80% of all lung cancer cases. The main reason of lung cancer‐related deaths is due to tumor metastasis. But, the mechanisms of NSCLC metastasis remains poorly understood. LncRNAs play pivotal roles in multiple biological processes. LncRNA‐HIT (HOXA transcript induced by TGF *β*) was recently identified. LncRNA‐HIT promotes cell migration, invasion, tumor growth, and metastasis. However, the detailed role of *lncRNA‐HIT* in NSCLC remains unknown. In this study, for the first time, we revealed a novel role of *lncRNA‐HIT* in the migration and invasion of NSCLC cells. The expression of *lncRNA‐HIT* was significantly upregulated in NSCLC tissues and cell lines, and the expression level of *lncRNA‐HIT* correlates with advanced disease stage and predicts unfavorable prognosis of NSCLC patients. Functional assays demonstrated that *lncRNA‐HIT* markedly increased the ability of NSCLC cells to migrate and invade. Furthermore, the molecular mechanism by which *lncRNA‐HIT* affects NSCLC cells was associated with regulation of ZEB1 stability. LncRNA‐HIT functions as a prometastasis oncogene by directly associating with ZEB1 to regulate NSCLC. The interaction of *lncRNA‐HIT* and ZEB1 may be a potential target for NSCLC therapy.

## Introduction

Lung cancer is the most common solid tumor and the leading cause of cancer‐related mortality worldwide 1, 2.Non‐small cell lung cancer (NSCLC), including squamous cell carcinoma, adenocarcinoma, adenosquamous cell carcinoma, and large cell carcinoma, accounts for approximately 80% of all lung cancer cases 3. Despite the recent advancements of the diagnosis and treatment of NSCLC, the prognosis of patients with lung cancer remains poor 4. The main reason of lung cancer‐related deaths is due to tumor metastasis 5. However, the invasion and metastasis of NSCLC greatly limit treatment options, and no effective therapy for NSCLC patients with distant metastasis is currently available. Therefore, understanding the mechanisms of NSCLC metastasis and identification of novel therapeutic targets are urgently needed.

Long noncoding RNAs (lncRNAs) are defined as a set of RNAs larger than 200 nt in length and without protein‐coding potential. LncRNAs play pivotal roles in multiple biological processes, such as chromatin remodeling, transcriptional control, posttranscriptional regulation, and intercellular signaling 6, 7. A growing number of studies have reported that the dysregulation of lncRNAs expression was involved in many cancers including lung cancer 8, 9. Some lncRNAs have been demonstrated to play important roles in lung carcinogenesis and have emerged as biomarkers for tumor diagnosis and prediction of prognosis 10, 11, 12.

LncRNA‐HIT (HOXA transcript induced by TGF*β*) was recently identified 13. LncRNA‐HIT regulates TGF‐*β*‐induced epithelial‐to‐mesenchymal transition (EMT). Knockdown of *lncRNA‐HIT* resulted in decreasing of cell migration, invasion, tumor growth, and metastasis 13. However, the detailed role of *lncRNA‐HIT* in NSCLC remains unknown. In this study, we elucidated the clinical significance of *lncRNA‐HIT* on the prognosis and clinicopathological characteristics of NSCLC patients. We also focused on the underlying molecular mechanisms by which *lncRNA‐HIT* promotes migration and invasion of NSCLC cells.

## Material and Methods

### Cell culture and samples

Five NSCLC cell lines (SK‐MES‐1, NCI‐H1650, A549, NCI‐H1975, 95D) and normal lung epithelial cells (NLEC) were purchased from ATCC. The cells were cultured in Dulbecco's modified eagle's medium (DMEM) containing 10% fetal bovine serum (FBS) at 37°C in 5% CO2. The tumor tissues and patient data were obtained from The Second Hospital of Jilin University. All of the patients were provided written informed consent.

### Viral infections

LncRNA‐HIT cDNA was cloned into the pLVX lentiviral vector (Addgene, Cambridge, MA). Virus was made using Turbofect transfection (Thermo, Boston, MA) into 293T cells. Virus was filtered and then infected into cells with polybrene for 24 h. Cells were selected with 3 *μ*g/mL puromycin (Invitrogen, Carlsbad, CA). The pLKO.1 shRNA lentiviral system was used to knockdown genes of interest. pLKO.1, pLKO.1‐shHIT‐1, and pLKO.1‐shHIT‐2 were purchased from Genechem Company, Shanghai, China. Target sequences for shRNAs are as follows: shHIT‐1: GTCTCACATACCTTCCTAACTCTAG, shHIT‐2: CCTCCAAGGTGGTCTGTGACCTTAA. Puromycin at 3 *μ*g/mLwas used to select stable cells.

### Western blot

Western blotting was performed as described previously 14. Primary antibodies 1:1000 anti‐ZEB1 (Abcam), 1:1000 anti‐E‐cadherin (CST),1:1000 anti‐ZO1 (CST), 1:1000 anti‐N‐cadherin (CST), 1:1000 antivimentin (CST), and 1:10000 anti‐GAPDH (Santa Cruz) were used. Secondary detection antibodies were anti‐mouse IgG‐HRP conjugate (Jackson) used at 1:10000 or anti‐rabbit IgG–HRP conjugate (Jackson). Blots were developed with ECL substrate (Millipore Boston, MA) and analyzed on an imager (GE Healthcare, London, UK).

### Migration and invasion assays

A total of 5 × 10^4^ cells were seeded in a serum‐free DMEM in the upper chamber of a 24‐well transwell migration or invasion insert (BD biosciences, Franklin lakes, NJ). The lower chamber was filled with medium containing 20% FBS. After 24 h of culture at 37°C, cells in the upper chamber were removed, and the cells that had traversed the membrane were fixed in 4% paraformaldehyde, and then stained by crystal violet.

### RNA isolation and quantitative real‐time PCR (qRT‐PCR)

Total RNAs of cells or tissue samples were isolated using TRIzol (Invitrogen) according to the manufacturers’ instructions. First‐strand cDNA was generated using the Reverse Transcriptase (Transgene, Beijing, China). qRT‐PCR was performed in the ABI 7500 Real‐Time PCR System using SYBR Green Mixture (Takara, Dalian, China). Data were normalized to GAPDH or to control samples. Primers sequences for the target genes were as follows: *lncRNA‐HIT*‐F: 5′‐TGAAA_GGGAGAGAAAGGAAAGG‐3′, *lncRNA‐HIT*‐R: 5′‐GACAGTCTAGGCATTGCTGAT‐3′; ZEB1‐F: 5′‐CCCAGGACAGCACAGTAAAT‐3′, ZEB1‐R: 5′‐GATGGTGTACTACTTCTGGAACC‐3′.

### RNA immunoprecipitation assay

RNA immunoprecipitation assay (RIP) assays were performed as described previously 15. RIP products were analyzed by qRT‐PCR. A total quantity of 5 *μ*g Snail1 (Abcam), Snail2 (Abcam), ZEB1 (Abcam), ZEB2 (Abcam), Twist1 (Abcam), or Twist2 (Abcam) antibodies were used for RIP reaction.

### Chromatin immunoprecipitation assay (ChIP)

ChIP assays were performed as described previously 16. The primers for the *CDH1* promoter were as follows: 5′‐ACTCCAGGCTAGAGGGTCACC‐3′ (sense) and 5′‐CCGCAAGCTCACAGGTGCTTTGCAGTTCC‐3′ (antisense).

### RNA pull‐down assay

RNA pull‐down and deletion mapping were performed as described previously 15. Briefly, biotin‐labeled *lncRNA‐HIT* were in vitro transcribed with the Biotin RNA Labeling Mix (Roche, Cambridge, UK) and T7 RNA polymerase (Roche), treated with RNase‐free DNase I (Roche), and purified with the RNeasy Mini Kit (Qiagen, Dusseldolf, Germany). Cell nuclear proteins were extracted using the Cytoplasmic and Nuclear Protein Extraction Kit (Tiangen, Beijing China). Cell nuclear extract was then mixed with biotin‐labeled *lncRNA‐HIT*. Washed streptavidin agarose beads (Invitrogen) were added to each binding reaction and further incubated at room temperature. Beads were washed briefly five times and boiled in sodium dodecyl sulfate buffer, and the retrieved protein was detected by the standard western blotting.

### Statistics

All experiments were repeated at least three times. Results are expressed as mean ± SD as indicated. A two‐tailed Student's *t* test was used for intergroup comparisons. *P* < 0.05 was considered statistically significant.

## Results

### *lncRNA‐HIT* is upregulated in NSCLC tissues and cell lines

**Figure 1:**
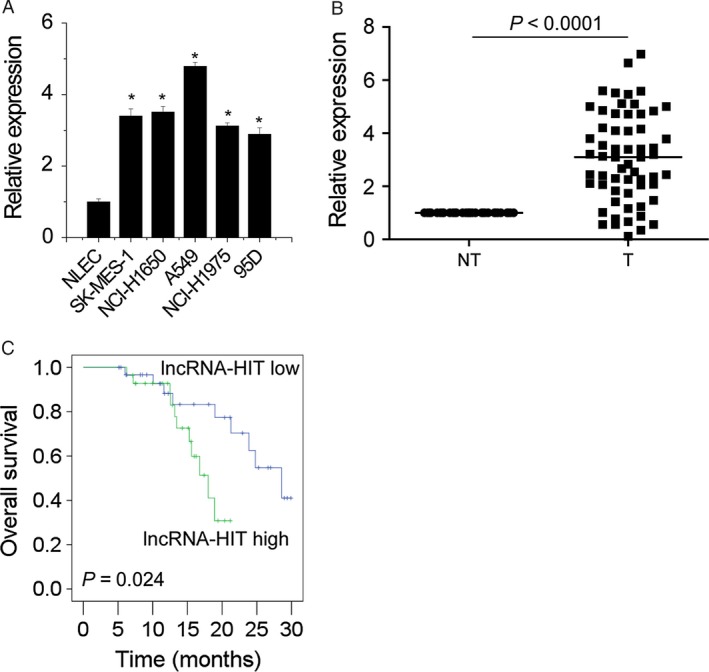
*lncRNA‐HIT* is upregulated in NSCLC tissues and cell lines and correlates with prognosis.(A) The relative expression of *lncRNA‐HIT* in different NSCLC cell lines and normal lung epithelial cells (NLEC). (B) The relative expression of *lncRNA‐HIT* in NSCLC tumor tissues (T) compared to matched adjacent nontumor tissues (NT) from 60 NSCLC patients. (C) Kaplan–Meier analyses of the correlations between lncRNA‐HIT expression level and overall survival of 60 patients with NSCLC. The cut point of high and low LncRNA‐HIT expressers is the median. Data are shown as mean ± SD. *P <* *0.05

We first examined the expression level of *lncRNA‐HIT* in NSCLC tissues and cell lines using qRT‐PCR. The results showed that the expression of *lncRNA‐HIT* of five NSCLC cell lines, namely SK‐MES‐1, NCI‐H1650, A549, NCI‐H1975, and 95D, was significantly higher than normal lung epithelial cells (NLEC) (Fig. 1A). In parallel, *lncRNA‐HIT* expression was markedly increased in NSCLC tumor tissues (T) compared to matched adjacent nontumor tissues (NT) from 60 patients with NSCLC (*P*<0.001) (Fig. 1B). Taken together, these data suggest that *lncRNA‐HIT* expression is upregulated in NSCLC.

### High‐level expression of *lncRNA‐HIT* correlates with advanced disease stage and predicts unfavorable prognosis

To investigate the clinical significance of *lncRNA‐HIT* in NSCLC, the correlation between *lncRNA‐HIT* expression and clinicopathological features were analyzed (Table 1). LncRNA‐HIT expression significantly correlated with the clinical staging (*P *=* *0.02) and distant metastasis (*P *=* *0.002). Furthermore, Kaplan–Meier analysis showed that NSCLC patients with high‐level *lncRNA‐HIT* expression group had a shorter median survival time than those in the low‐level group (Fig. 1C). These results suggested that *lncRNA‐HIT* expression may be a novel valuable marker for the prognosis of NSCLC patients.

**Table 1:** The relationship between *lncRNA‐HIT* expression and the clinicopathological characteristics of Non‐Small Cell Lung Cancer (NSCLC) patients

Features	*lncRNA*‐*HIT*		*P*‐value
	Low	High	
Age			
≤60	23	21	0.559
>60	7	9	
Gender			
Male	24	23	0.754
Female	6	7	
Histology			
Squamous cell carcinoma	10	9	0.898
Adenocarcinoma	13	12	
Adenosquamous carcinoma	2	3	
Bronchioalveolar carcinoma	5	6	
Clinical stage			
I, II	20	11	0.02
III, IV	10	19	
Distant metastasis			
No	21	9	0.002
Yes	9	21	
Drug/radiation therapy			
No	9	10	0.781
Yes	21	20	

*P‐*values were derived with a two‐sided Pearson chi‐square test.

### Silence of *lncRNA‐HIT* inhibits the migration and invasion of NSCLC cells

**Figure 2:**
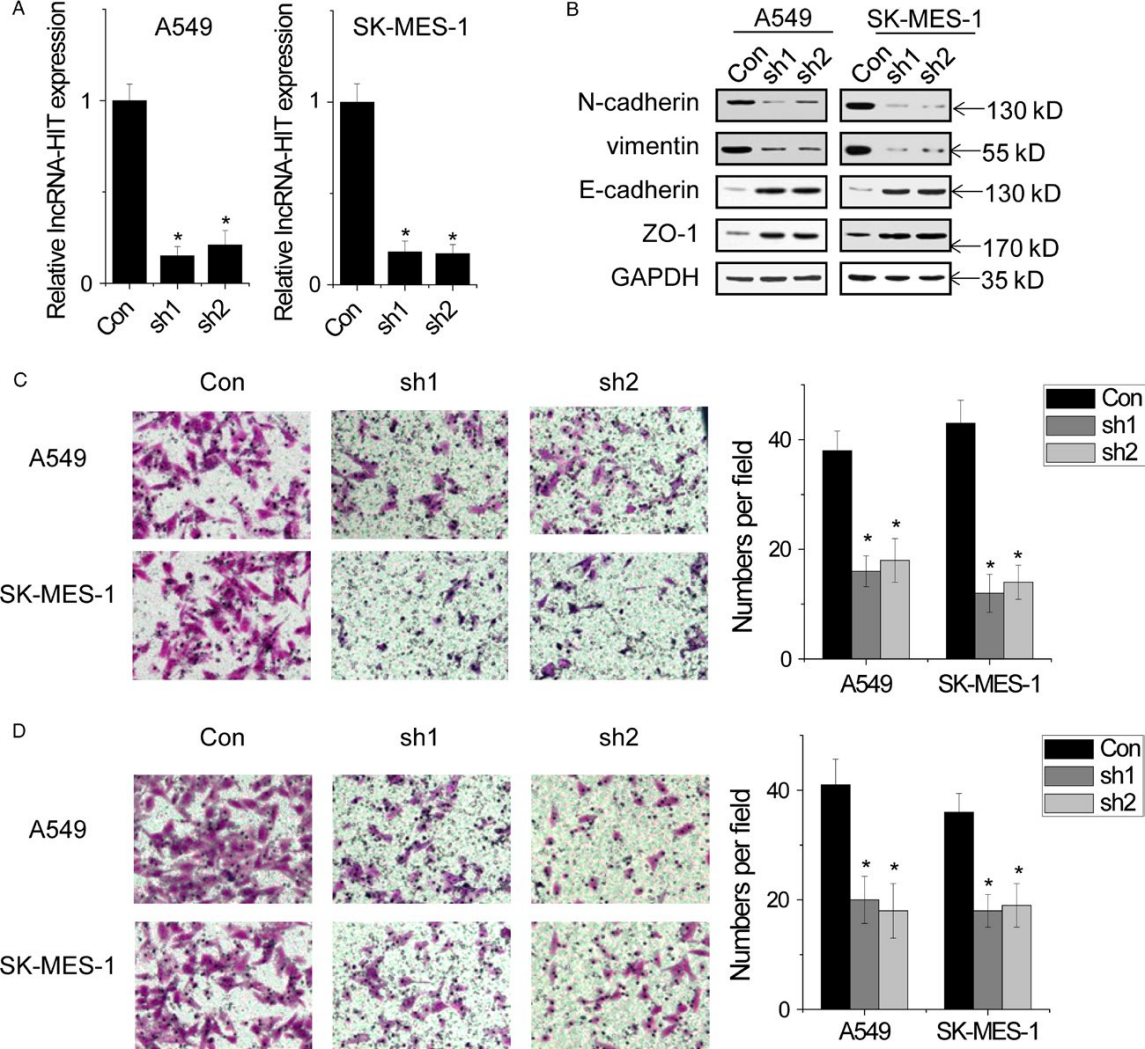
Silence of *lncRNA‐HIT* inhibits the migration and invasion of NSCLC cells. (A) The relative expression of *lncRNA‐HIT* in control and *lncRNA‐HIT* knockdown cells. (B) The EMT markers detected by western blot in control and *lncRNA‐HIT* knockdown cells. (C) Knockdown of *lncRNA‐HIT* suppressed migration in NSCLC cells. (D) Knockdown of *lncRNA‐HIT* suppressed invasion in NSCLC cells. Data are shown as mean ± SD . *P <* *0.05. EMT, epithelial‐to‐mesenchymal transition.

Based on the above results, we hypothesized a relationship between *lncRNA‐HIT* and NSCLC cell migration and invasion. To determine the role of *lncRNA‐HIT* in NSCLC cell migration and invasion, we silenced *lncRNA‐HIT* expression using lentivirus‐mediated shRNAs in A549 and SK‐MES‐1 cells (Fig. 2A). Notably, *lncRNA‐HIT* depletion significantly reduced the expression of mesenchymal markers, N‐cadherin and vimentin, and increased the expression of epithelial markers, E‐cadherin and ZO‐1 (Fig. 2B). The migration assay showed that *lncRNA‐HIT* knockdown inhibited the migratory capabilities of both A549 and SK‐MES‐1 cells compared to their control cells (Fig. 2C). Moreover, *lncRNA‐HIT*‐silenced NSCLC cells showed much weaker abilities to invade through Matrigel than did control cells (Fig. 2D).

### Overexpression of *lncRNA‐HIT* promotes the migration and invasion of NSCLC cells

**Figure 3:**
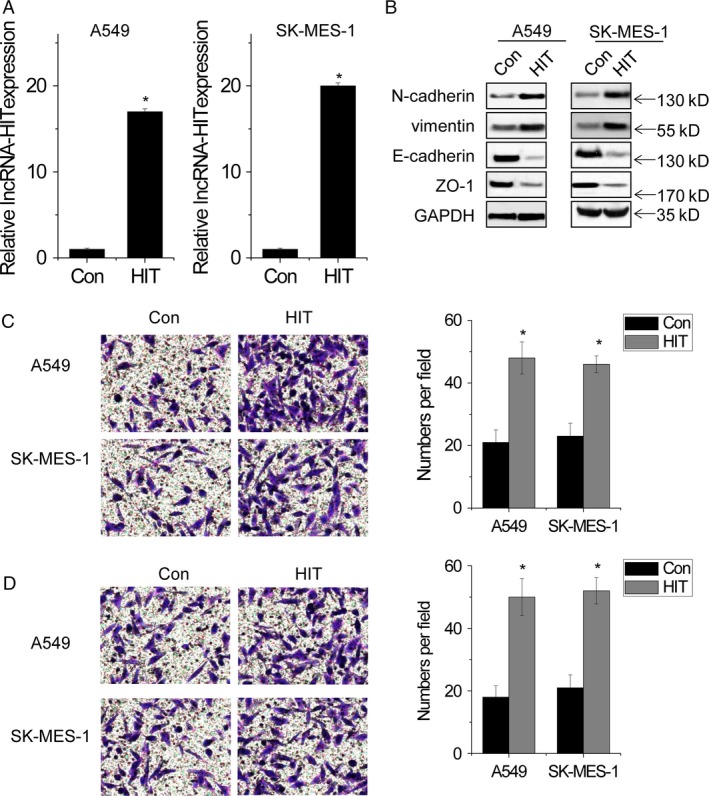
Overexpression of *lncRNA‐HIT* promotes the migration and invasion of NSCLC cells. (A) The relative expression of *lncRNA‐HIT* in control and *lncRNA‐HIT* overexpressed cells. (B) The EMT markers detected by western blot in control and *lncRNA‐HIT* overexpressed cells. (C) Overexpression of *lncRNA‐HIT* promoted migration in NSCLC cells. (D) Overexpression of *lncRNA‐HIT* promoted invasion in NSCLC cells. Data are shown as mean ± SD. *P <* *0.05. EMT, epithelial‐to‐mesenchymal transition.

In contrast, gain‐of‐function of *lncRNA‐HIT* with lentivirus‐mediated overexpression was performed to evaluate whether ectopic *lncRNA‐HIT* expression promotes the invasiveness of NSCLC cells (Fig. 3A). Overexpression of *lncRNA‐HIT* significantly inhibited the expression of epithelial markers, E‐cadherin and ZO‐1, and increased the expression of mesenchymal markers, N‐cadherin and vimentin (Fig. 3B). In addition, overexpression of *lncRNA‐HIT* obviously promoted the migratory and invasive abilities of both A549 and SK‐MES‐1 cells (Fig. 3C and 3D). Taken together, these data demonstrated that *lncRNA‐HIT* promotes EMT and remarkably induces the invasive phenotype of NSCLC cell lines.

### LncRNA‐HIT associates with ZEB1

**Figure 4:**
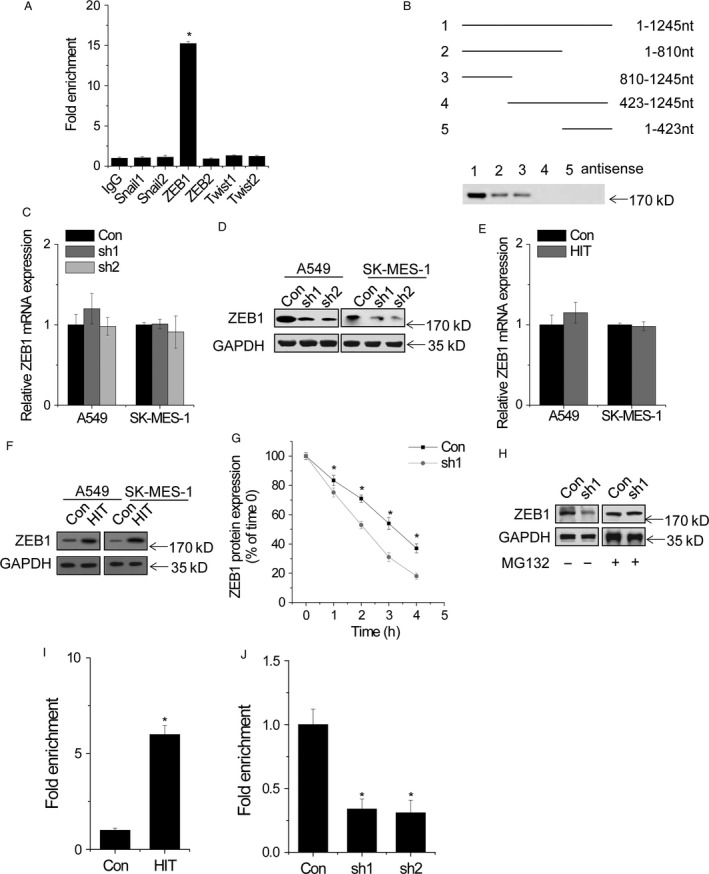
*lncRNA‐HIT* associates with ZEB1. (A) RIP assays for *lncRNA‐HIT* were performed using indicated antibodies. (B) Deletion mapping of ZEB1‐binding domain in *lncRNA‐HIT* (Up). Western blot for ZEB1 in samples pulled down by different *lncRNA‐HIT* fragments (Down). Antisense *lncRNA‐HIT* was used as a negative control. (C) The relative ZEB1 mRNA expression in control and *lncRNA‐HIT* knockdown cells. (D) The ZEB1 protein level in control and *lncRNA‐HIT* knockdown cells. (E) The relative ZEB1 mRNA expression in control and *lncRNA‐HIT* overexpressed cells. (F) The ZEB1 protein level in control and *lncRNA‐HIT* overexpressed cells. (G) The stability of ZEB1 protein over time was measured by western blot relative to time 0 after blocking new protein synthesis with 100 mg/ml CHX in control and *lncRNA‐HIT* knockdown A549 cells. (H) ZEB1 protein expression in control and *lncRNA‐HIT* knockdown A549 cells treated with vehicle control (DMSO) or 10 *μ*mol/L MG132 for 12 h. (I) The occupancy of ZEB1 in the promoter of *CDH1* was measured by ZEB1 ChIP assay followed by qRT‐PCR in control and *lncRNA‐HIT* knockdown A549 cells. (J) The occupancy of ZEB1 in the promoter of *CDH1* was measured by ZEB1 ChIP assay followed by qRT‐PCR in control and *lncRNA‐HIT* overexpressed A549 cells. Data are shown as mean ± SD. *P <* *0.05. RIP, RNA immunoprecipitation assay

Suppression of E‐cadherin, induced by Snail, ZEB, or Twist family, is considered not only a hallmark of EMT but also a key driver of EMT and metastasis 17. We suspected that whether *lncRNA‐HIT* regulates E‐cadherin expression through association with these EMT‐related transcription repressors. We detected the association between *lncRNA‐HIT* and these repressors by performing RIP assays. The results showed that *lncRNA‐HIT* was significantly enriched by ZEB1 antibody than the nonspecific IgG control antibody, Snail1, Snail2, ZEB2, Twist1, or Twist2 antibody (Fig. 4A). To validate the association and determine the specific binding region between *lncRNA‐HIT* and ZEB1, we performed RNA pull‐down assay and deletion‐mapping experiments. We found a 423‐nt region at the 5′ end of *lncRNA‐HIT* required for the association with ZEB1 (Fig. 4B). Taken together, we demonstrated a specific association between *lncRNA‐HIT* and ZEB1.

Next, we determined the function of *lncRNA‐HIT*‐ZEB1 association. Silence of *lncRNA‐HIT* significantly suppressed the protein level of ZEB1, but had no effect on the ZEB1 mRNA level (Fig. 4C and D). In contrast, overexpression of *lncRNA‐HIT* increased the protein level, but not mRNA level, of ZEB1 (Fig. 4E and F). These results strongly indicated that the association of *lncRNA‐HIT* and ZEB1 may influence the stability of ZEB1 protein. To further confirm the *lncRNA‐HIT*‐mediated ZEB1 regulation, we treated control and *lncRNA‐HIT* knockdown A549 cells with cycloheximide (CHX). We found that the half‐life of ZEB1 was much shorter in *lncRNA‐HIT* knockdown A549 cells than that in control cells (Fig. 4G). When MG132, an inhibitor of proteasome degradation was used, the ZEB1 protein level in *lncRNA‐HIT* knockdown A549 cells was markedly upregulated and reached a level that was comparable to that in control cells (Fig. 4H). Taken together, these data suggested that *lncRNA‐HIT* is important for the stability of ZEB1 protein.

Next, we examined whether *lncRNA‐HIT* affected ZEB1 occupancy of the promoter region in *CDH1*. The effect of *lncRNA‐HIT* on the ZEB1 occupancy of *CDH1* promoter was evaluated using a ChIP assay followed by qPCR. We found that overexpression of *lncRNA‐HIT* significantly increased the occupancy of ZEB1 on the promoter region of *CDH1*, while silence of *lncRNA‐HIT* reduced the binding of ZEB1 on the *CDH1* promoter (Fig. 4I and J).

### LncRNA‐HIT promotes migration and invasion via regulation of ZEB1 expression

**Figure 5:**
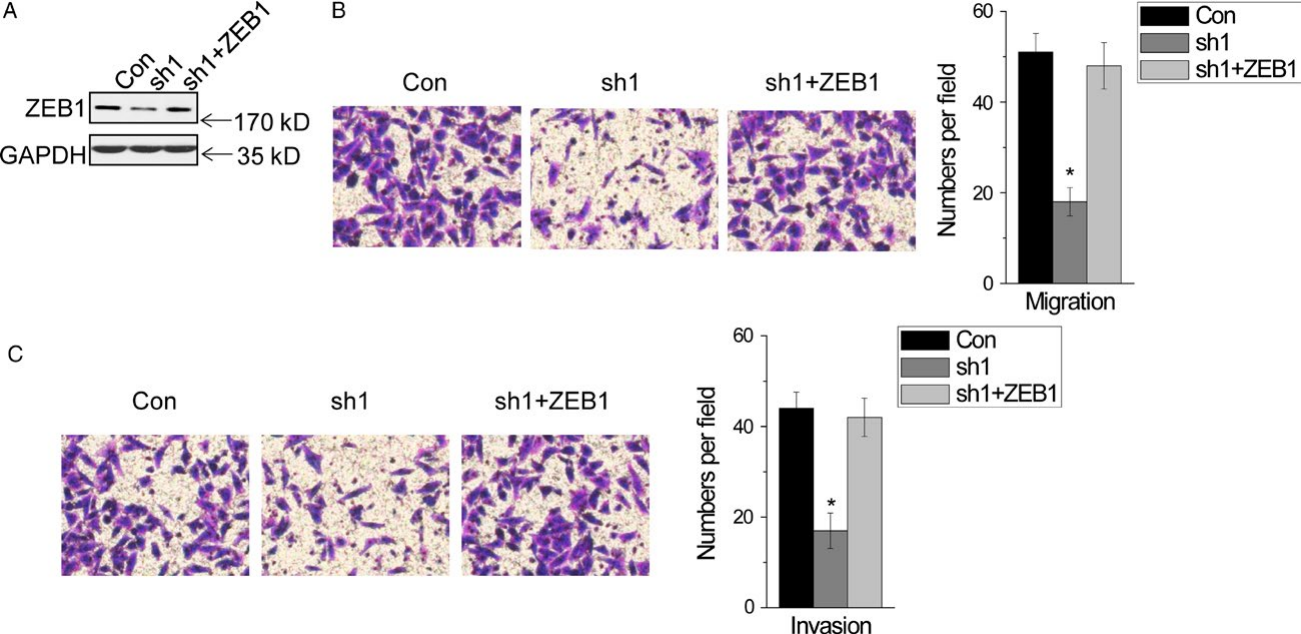
*lncRNA‐HIT* promotes migration and invasion via regulation of ZEB1 expression. (A) ZEB1 protein expression in A549 control and *lncRNA‐HIT* knockdown cells expressing control and ZEB1. (B) Migration of A549 cells expressing control and *lncRNA‐HIT* shRNAs with and without ZEB1. (C) Invasion of A549 cells expressing control and *lncRNA‐HIT* shRNAs with and without ZEB1. Data are shown as mean ± SD. *P <* *0.05.

Finally, we determined whether *lncRNA‐HIT* promotes migration and invasion via regulation of ZEB1 expression. We found that overexpression of ZEB1 (Fig. 5A) significantly rescued the cell migration and invasion phenotypes induced by *lncRNA‐HIT* knockdown (Fig. 5B and C). Together, these results demonstrate that *lncRNA‐HIT* exerts its function at least in part through regulating ZEB1 expression.

## Discussion

In this study, for the first time, we revealed a novel role of *lncRNA‐HIT* in the migration and invasion of NSCLC cells. The expression of *lncRNA‐HIT* was significantly upregulated in NSCLC tissues and cell lines, and the expression level of *lncRNA‐HIT* correlates with advanced disease stage and predicts unfavorable prognosis of NSCLC patients. Silence of *lncRNA‐HIT* effectively decreased the migration and invasion of NSCLC cells. In contrast, the overexpression of *lncRNA‐HIT* markedly increased the ability of NSCLC cells to migrate and invade. The molecular mechanism by which *lncRNA‐HIT* affects NSCLC cells was associated with regulation of ZEB1 stability. *lncRNA‐HIT* may function as a prometastasis oncogene by directly associating with ZEB1 to regulate NSCLC.

Many large‐scale sequencing studies have demonstrated that >9000 genomic loci express lncRNAs 18, 19. However, the vast majority of these lncRNAs remain functionally uncharacterized in NSCLC. Increasing evidence suggests that lncRNAs play important roles in oncogenesis and metastasis in NSCLC. For example, lncRNA AGAP2‐AS1 promotes NSCLC growth through interacting with EZH2 and LSD1 and repressing LATS2 and KLF2 expression 8. LncRNA TATDN1 promotes NSCLC metastasis through suppression of E‐cadherin 20. These and other examples suggest that lncRNAs are critical to many oncogenic processes. *lncRNA‐HIT*, an oncogene, has been documented to promote tumor metastasis in breast cancer 13. However, the role of *lncRNA‐HIT* in NSCLC remains unclear. We first analyzed the expression of *lncRNA‐HIT* on the NSCLC tissues cell lines. LncRNA‐HIT was significantly upregulated, correlates with advanced disease stage, and predicts unfavorable prognosis, indicating that *lncRNA‐HIT* may play an important role in NSCLC invasion and metastasis.

Functional assays demonstrated an important role of *lncRNA‐HIT* in NSCLC progression, including migration and invasion. Mechanistic study revealed that *lncRNA‐HIT* exerts prometastasis function at least in part through association with ZEB1. ZEB factors contain multiple domains to interact with other transcription factors, which is essential for regulation of EMT. For example, ZEB proteins can recruit histone deacetylases, methyltransferases, and polycomb group proteins 21, 22, 23. ZEB1 also suppressed transcription through recruitment of the SWI/SNF chromatin remodeling ATPase BRG1 24. Recent studies have found a set of lncRNA that can regulate ZEB1 expression through sponging miRNAs, such as lncRNA‐ATB and lncRNA‐NEAT1 25, 26. In this study, for the first time, we revealed a novel posttranslational manner, in which lncRNA regulates ZEB1. We found that *lncRNA‐HIT* could evaluate the stability of ZEB1 and then increase the ZEB1 occupancy of the promoter region in *CDH1*. The interaction of *lncRNA‐HIT* and ZEB1 may be a potential target for NSCLC therapy.

Overall, our study is the first to show that *lncRNA‐HIT* plays an important role in metastasis in NSCLC. LncRNA‐HIT promotes cell migration and invasion at least in part through interaction with ZEB1 regulating its expression. However, it should be pointed out that our in vitro observations may not be completely applied to in vivo situations in the absence of in vivo studies. The possible role of *lncRNA‐HIT* in NSCLC required further studies using animal models of tumors.

## Conflict of Interest

None declared.
